# Interventions supporting community nurses in the provision of Oral healthcare to people living at home: a scoping review

**DOI:** 10.1186/s12912-022-01051-5

**Published:** 2022-10-05

**Authors:** Patrick Stark, Gerry McKenna, Christine Brown Wilson, Georgios Tsakos, Paul Brocklehurst, Caroline Lappin, Barry Quinn, Gary Mitchell

**Affiliations:** 1 School of Nursing and Midwifery, Queen’s University Belfast, Belfast, Northern Ireland; 2 Centre for Public Health, Belfast, Queen’s University Belfast, Belfast, Northern Ireland; 3 Department of Epidemiology and Public Health, University College London, London, UK; 4 School of Health Sciences, Bangor University, Bangor, UK; 5 Department of Health, Castle Buildings, Stormont, Belfast, Northern Ireland

**Keywords:** Oral health, Oral care, Community nurses, Community nursing, Home care, Scoping review, Interventions

## Abstract

**Background:**

Oral health is a critical issue for public health and poor oral health is associated with significant chronic health conditions and lower quality of life. There has been little focus on providing oral health care to people who receive care in their own homes, despite the high risk of poor oral health in older people. Nurses practicing in the community are well placed to deliver this care, but little is known about how to build this capability through education or training interventions.

**Methods:**

A scoping review methodology was employed to find and review studies of oral health interventions involving populations of people receiving care in their own home or those nurses who deliver this care. The research question asked what previous research tells us about oral health interventions delivered by nurses in the community. Data was extracted for four areas: setting and type of intervention, patient outcomes, changes to nursing practice and implementation and process evaluations of interventions.

**Results:**

Two thousand eighty papers were found from the searches, and only nine were ultimately deemed eligible for inclusion in the review. Included studies spanned community nursing for older people (*n* = 3) and health visiting or community nursing for children and infants (*n* = 6). Patient outcomes were generally positive, but this is based on a low level of evidence. Changes to practice including increased oral health care administered by nurses were found, but this required professional support to be sustainable.

**Conclusions:**

This review has found that there is a clear gap in the research around interventions designed to be used by community nurses to improve oral health care for people receiving care in their own homes. The results also suggest that any future intervention must make use of a participatory, co-design approach and consider the complex setting of nursing practice in the community and the barriers to delivering this care, such as time pressure and lack of prior experience.

## Background

Oral health is an important measure of a person’s overall health and wellbeing [1]. The mouth and teeth are an integral part of the body and enable essential human development and function across the lifespan including the activities of eating, smiling and talking [2]. Despite its importance, oral conditions are a global public health challenge [3, 4]. Although mostly preventable, these health conditions affect approximately 3.5 billion people worldwide [4] (including dental caries, periodontal (gum) disease, tooth loss and oral cancer [5, 6]). Oral health affects a person’s general health as poor oral health can lead to mouth pain, discomfort and inability to chew food properly [7]. Chronic oral health conditions are also associated with chronic systemic health problems, including frailty, diabetes, ischaemic heart disease, renal disease and respiratory disease [8–11]. While oral healthcare is often discussed as a key priority, it often remains a neglected issue and is rarely seen as a priority in current health policy [12, 13].

As the largest body of professional healthcare providers, nurses have the potential to occupy an important role in providing health promotion about oral health. Nurses practice in a context where it is possible to conduct structured oral health assessments and implement practices to optimise the care of people’s oral health [14–16]. Despite this, literature suggests that many nurses still lack confidence in both undertaking structured oral health assessments and promoting good oral health practice to patients [17–20].

A recent report by the World Health Organisation [21] estimated that several million people received care in their own homes. Many of these people also have complex care needs and rely on community nurses to provide effective support in the management of their care [22–24]. These individuals are at a high risk of developing poor oral health and current NICE [25] guidance recommends that health and social care services need to provide support, in the form of health promotion, assessment and care-planning, to these people at risk. Community nurses are frequently the first point of contact with these patients, and as such, it is important that they have requisite knowledge and competence to support patients in their own home to maintain good oral healthcare [26–28]. However, the use of specific tailored approaches to support the oral healthcare of people living at home requiring community nursing support remains unclear [29]. It is therefore important to establish which current evidence-based interventions exist that may support community nurses in optimising the oral care of people they are supporting to live at home.

The aim of this scoping review is to examine current interventions that support community nurses in the provision of oral healthcare to people living at home. Within this aim, any intervention that supports community nurses across the lifespan will be considered. For example, those who typically deal with older people (e.g., district nurses) and nurses who visit children and infants (e.g., health visitors) in their own homes will be examined. Beyond this immediate aim of the review, this research aims to inform the design of a digital educational resource to be used by community nurses who care for people in their own home.

## Methods

### Methodological framework

This scoping review followed the Arksey and O’Malley [30] methodological framework with further guidance from the Colquhoun et al. [31] commentary on scoping review reporting standards, the PRISMA guidelines for scoping reviews [32] and Levac et al.’s scoping review methodology paper [33]. The methodological stages of the Arksey and O’Malley framework followed were: 1) Identifying the research question, 2) Identifying relevant studies, 3) Study selection, 4) Charting the data, 5) Collating, summarizing and reporting results.

### Identifying the research question

The intended outcome of the review was considered in detail, as recommended by Levac et al. [33] The contextual rationale of this research is to use the research results to support the co-design of a digital educational intervention for community nurses to improve their delivery of oral health care. A priority for the scoping review, therefore, was that it needed to include the findings of past research into oral health interventions that involved community nurses. Although the professional context of community nurses in the UK is that they typically care for older people, we expected that research into interventions involving health visiting for children and infants could be equally informative in terms of professional training and facilitators and barriers to improving nurses’ knowledge and delivery of oral health care. Our research question, therefore, encompasses community nurses across the lifespan.

The primary research question was: “What does previous research tell us about oral health interventions delivered by nurses in the community?”. Specifically, we aimed to investigate four main areas: setting and type of intervention, patient outcomes, changes to nursing practice and implementation and process evaluations of interventions. Setting and type of intervention was investigated to ensure an understanding of the professional context and nature of the intervention. Patient outcomes were investigated as this review was conducted within the context of designing a new intervention and it is important to consider the past success of similar interventions. Changes to practice were investigated as this is arguably the most important intermediate outcome of any practice-based intervention. Changes to practice such as changes to oral health care skills, oral health care planning and frequency of delivery of oral health care were examined. Whilst the ideal distal goal is an improvement in patient outcomes, these more proximal and nurse-focussed outcomes were investigated to examine if the reviewed interventions showed promise in increasing nurse capability and if it was applied in practice. Finally, implementation and process evaluation data were investigated to explore facilitators and barriers of intervention delivery.

### Identifying relevant studies

We searched CINAHL, Embase, Medline and PsycINFO. We did not apply any time frame limitations to the searches. The search terms were developed by using free text terms which related to people living in the community, community nurses and oral health. Free text terms were entered individually into the search functions of each database to allow medical subject headings (MeSH terms) to be selected where available. MeSH terms such as “Community Health” and “Community health nursing” were used and inclusive of both community nursing for older people and of health visiting for children and infants. A search term for specialist nursing was included due to community nursing being a speciality in its own right, and to capture papers where other types of specialist nurses, for example palliative care nurses, were practicing in the community. Truncation (*) was used to capture variations of search terms. An example set of search terms from CINAHL is available in Table 1. The search was conducted in May 2021, after which point the process of study selection began. Later in the project, a search was repeated to capture any additional studies published between May 2021 and December 2021 which meet the inclusion criteria, but none were eligible.

**Table 1:** Example search terms for CINAHL

Component of research question	Search terms
People living in the community	((“Community Setting”) OR “Elderly” OR “Home” OR (“Group Home”) OR ((MH “Assisted Living”)) OR (“Supported Living*”) OR (“Supported Hous*”) OR “Independent*” OR “Shelter*”)
Community nurses	AND ((“Community nurs*”) OR ((MH “Community Health Nursing”)) OR (“Primary Care Nurs*”) OR (“District nurs*”) OR (“Community palliati*”) OR (“specialist nurs*”) OR (“community health*”) OR (“primary health*”) OR (“Public health*” OR (MH “Public Health”)))
Oral health	AND (“Oral*” OR “Dental*” OR (“Dry mouth”) “Carie*” OR ((MH “Tooth”) OR “Tooth*”) OR “Edentulism*” OR “Periodontal*” OR ((MH “Xerostomia”)) OR (“Broken teeth”) OR (“broken tooth”) OR (“missing teeth”) OR (“missing tooth”) OR (“cancer oral”) OR (“cancer of mouth”) OR (“symptoms of oral*”) OR ((MH “Gingivitis”)) OR (“Bleeding gum*”) OR “gum*” OR “pyorrhea*”)

### Study selection

The database results were exported to Covidence [34] and all abstracts were screened by two members of the research team (PS & GM) for inclusion or exclusion in full text review. A third team member (GMcK) was available should consensus not be reached for inclusion or exclusion, but this was not required. The inclusion criteria were: “1) The population includes people receiving community nursing care or those who deliver this care. 2) The study describes an intervention for oral healthcare 3) The study design is randomised controlled trial (RCT), non-RCT, quasi-experimental, cross sectional, interrupted time series, controlled/uncontrolled before/after, case control, cohort, qualitative, scoping review or systematic review. For inclusion criterion 1, we did not use any age limit, i.e., people of any age who receive community nursing care were seen as an eligible population as were those registered nurses who deliver this care. The exclusion criteria were: “Reject study designs of case reports, case series or commentary.”

### Charting the data

After the initial screening, a data charting tool was developed to include detail on study location, design, population, intervention, methods, outcomes, analysis and results. As recommended by Levac et al. [33], the development of the charting strategy was an iterative process. The research team met after charting began and further detail on implementation and process evaluation was added to the charting tool. The decision to include data from previous interventions on facilitators and barriers to intervention engagement or successful outcomes was in consideration of the scoping review’s aim of informing the design of future oral health interventions. Data charting was undertaken by two research team members (PS & GM).

### Collating, summarizing and reporting the results

Results from the data charting process were collated into themes around implementation and process evaluation, changes to practice and patient outcomes data. As recommended by Levac et al. [33] these themes are discussed in the context of the intended outcome of the study, i.e., the informing of an oral health intervention to be delivered by community nurses. We have focused on an overview of the evidence, identifying key information that may inform intervention design rather than a critical appraisal of the evidence, which need not be the focus of a scoping review [35]. Although this scoping review is not concerned with providing high-security estimates of the efficacy of oral health education interventions, evidence of intervention success, i.e., patient outcome analysis, is helpful contextual information when reviewing information on design, implementation and changes to practice.

## Results

**Fig. 1:**
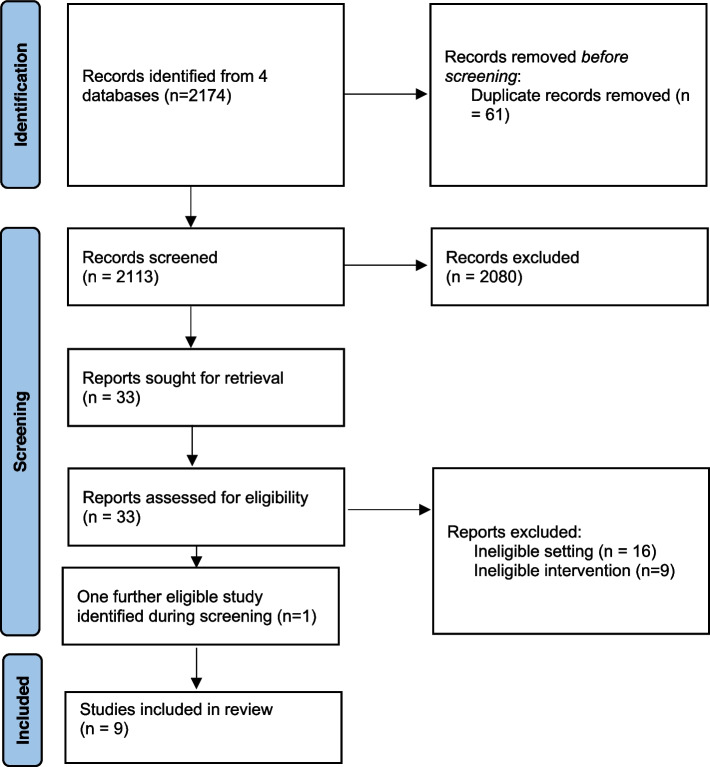
PRISMA flow diagram

A total of 2174 records from four databases were imported to Covidence [34] which automatically removed 61 duplicate records (Fig. 1). The abstract screening criteria were applied, and this resulted in 2080 records excluded Following title/ abstract screening with 33 papers moving to full text review. Resources were assessed for eligibility and a further 25 were excluded. These studies were excluded because they either did not report an eligible population (inclusion criterion 1) or eligible intervention (inclusion criterion 2). One further paper was found and deemed to be eligible when reviewing the reference lists of included studies. A total of 9 papers were included in the final review (Table 2).

**Table 2:** Scoping review of oral health education interventions in community nursing settings

Study	Design	Setting	Participants	Intervention	Methods	Outcomes	Analysis	Results	Implementation and process evaluation
Brickhouse, T.H.,Haldiman, R.R., Evani, B. (2013) [36] – USA	Propensity score matched (PSM) control	Health visiting for at-risk children and families	*N* = 216 children. Participated in CHIP Begin with a Grin. 6-36 months.	CHIP Begin with a Grin – Community health nurses and paediatric nurse practitioners applied fluoride dental varnish to the teeth of children aged 6-36 months not currently seeing a dentist. Educated primary caregiver on oral hygiene, nutrition and oral health literacy. Aimed to reduce childhood caries. Practiced these oral health techniques with nurse.	Created control group using PSM. 216 intervention, 216 control. Used Medicaid database to gather data and conduct a quasi-experimental study.	Dental utilization (number of claims for dental care)	Logistic regression	Significantly higher instances of making > 1 dental claim for intervention group. 3 times more likely to have at least one dental claim. Odds ratio: 3.0, confidence interval: CI: 1.9–4.7)	None reported.
Whittle, J.G., Whitehead, H.F., Bishop, C.M. (2008) [37]– England	Randomised control trial	Health visiting for children at home	251 control, 250 intervention, aged 3 years	No name stated - Dental health advice given to parents by health visitor. Leaflet “giving teeth a good start”, 440 pm fluoride toothpaste and child toothbrush also provided. Main messages were around how to avoid sugar, increase health eating, tooth brushing, educating children from infancy about oral health and selecting an appropriate toothbrush and toothpaste. Diet record sheets and advice given at 20 months, and more toothpaste and toothbrush.	Control and intervention samples were recruited at 8 months old, randomised using balanced blocks, stratified by ethnicity and location. First visit for intervention group was soon after recruitment, then again at 20 months. Sample tested at 3 years for decayed, missing and filled surfaces (DMFS), then again at 5 years. Also compared with large census data (*n* = 2253) at 5 years.	Teeth were examined using British Association for the Study of Community Dentistry survey items for decayed, missing and filled surfaces of teeth. Recorded number in these three categories.	Calculated 95% Cis for DMFS data and compared for significant differences.	No statistically significant differences were found between control and intervention groups, although the gap widened as the children grew older. A significant difference was found between the intervention group and the data for all 5-year-olds in the area, with intervention group having significantly less incidences of damaged, missing or filled surfaces in their teeth.	None reported.
Haber, J., Hartnett, E., Hille, A., Jessamin, C. (2020) [38] – USA	Quasi-experimental.	Nurse home visiting for first-time mothers	4 nurses in intervention plus support group, 10 nurses in a comparison group of intervention only with no support. 27 clients of intervention nurses (first time mothers).	Cavity Free Kids – Evidence based curriculum, Cavity Free Kids, was given to Nurse Family Partnership nurses (a not-for-profit program for nurse home visiting to low-income first-time mothers by a registered nurse). Cavity Free Kids is a 2-hour program to increase knowledge and practice behaviours of oral health. Aimed to increase integration of oral health into home visiting (from pregnancy to age 2).	N = 32 nurses received the oral health intervention, but only Miami based nurses were given instruction and support to integrate into practice. Non-Miami nurses formed a comparison group (but had received the original intervention). 4 Miami nurses completed survey data collection at baseline, 30 days and 90 days. 10 non-Miami nurses also completed data collection. 27 clients of Miami nurses were given a 10-item telephone questionnaire at baseline, 30 and 90 days.	Oral health knowledge and practice behaviours (nurses, self-report). Oral health child care behaviours, oral health behaviours, information received about oral health (clients).	Descriptive statistics to compare nurse groups, paired sample t-tests to measure change in client outcomes from baseline to later data collection points.	At 90-day follow-up, non-Miami nurses (no support) reported lower levels oral health practice behaviours than Miami nurses who received support, including: reporting of explanation to clients about how to brush children’s teeth and lower levels of referring 0–2-year-olds to a dentist, and discussion of how to prevent cavities in 0–2-year-olds. Miami clients reported increased levels of information received from nurses about oral health and increased cleaning of their child’s mouth. Clients who ‘graduated’ the full 2-year NFP program completed an additional survey – none reported any visible plaque, staining or decay on their child’s teeth (signs of ECCs).	100% of Miami nurses who received support were using the intervention in practice at 90 days. Only 30% of non-Miami nurses (no support) were using the intervention
Lewis, A., Kitson, A., Harvey, G. (2016) [39] – Australia	Pre-post implementation study	Home care	319 home care clients (aged 61-82)	Better Oral Health in Home Care (BOHHC) – multidisciplinary model incorporating oral health assessment, oral healthcare planning, actioning daily oral care, and referral for dental treatment. Training and skill development for home care workers.	Mixed methods. Analysed oral-health assessments of home care clients, self reported OH outcome data from pre and post BOHHC implementation. Pre and post training questionnaires. Dental referral statistics analysed for changes pre-post. Qualitative data from steering committee meetings, and project meetings.	Analysed oral-health assessments of home care clients, OHIP-14 – self reported OH outcome data from pre and post BOHHC implementation. Pre and post training questionnaires. Dental referral statistics also analysed. Qualitative data from steering committee meetings, and project meetings.	Comparison of pre and post means (McNemar tests). Thematic analysis for qual.	Oral Health assessment increased – no uptake at pre-test, four home care providers had uptake at post-test. Oral Healthcare planning also increased from zero home care providers to four. Increased staff confidence at delivering oral care. Increased self-reported oral health, but too much missing data to allow conclusion around reduction in oral disease.	Home care workers data revealed a ‘lone worker’ theme with considerable time pressure. Promoting a “stop, check and act” strategy was developed to help in this context. Implementation and process analysis – building relationships was a key to facilitating delivery. Cultural respect for Aboriginal community clients. Mentoring from project director. Universal integration of BOHHC model was a facilitator, alongside knowledge transfer (KT) with an assigned KT expert.
Lewis, A., Harvey, G., Hogan, M., Kitson, A. (2019) [40] – Australia	Realist evaluation	Home care	Twelve home care staff, two patients	Better Oral Health in Home Care (BOHHC) – A multidisciplinary model incorporating oral health assessment, oral healthcare planning, actioning daily oral care, and referral for dental treatment. Training and skill development for home care workers.	14 semi-structured interviews, face to face or phone. Two time points. One at point of implementation, a second five years’ post-implementation	Facilitators and barriers to intervention delivery, contextual characteristics which supported or undermined the embedding of the intervention.	Thematic analysis	Home care workers reported significantly improved OH knowledge and skills and this increased staff ability to recognise patients in need of OH support and dental referrals.	High level corporation engagement was achieved. Development of capacity building networks (internal and external) was seen as a facilitator. OH assessment tool was introduced. High staff turnover was a barrier.
A. Nihtilä, K. Komulainen, E. Tuuliainen, I. Nykänen, S. Hartikainen, A.L. Suominen. (2017) [41]– Finland	Non randomised population study with control and intervention	Home care	Patients receiving home care (*n* = 141 intervention, *n* = 108 control, aged 84.3, 84.6 years respectively)	Nutrition, Oral Health and Medication – NutOrMed – Tailored dietary and oral health intervention for home care clients 75 years or over. Individual counselling on dry mouth care. Advice on topical therapies. Nutrition intervention for patients at risk of malnutrition.	Interviews before and after a tailored intervention. In-home.	Xerostomia, liquid intake, dietary changes	Descriptive	Xerostomia decreased by 7.1%, liquid intake increased 21.6%, eating fruits and vegetables increased 10% in intervention group. Control group - 2.8% Increase in xerostomia, liquid intake decreased 5.4 and 2.1% increase in eating fruits and vegetables. Topical therapies alone not enough, interventions for nutrition stated to be particularly effective for xerostomia. Dietary changes noted to be effective for xerostomia (but no evidence stated for the breakdown of these conclusions).	None reported.
Nihtilä A, Tuuliainen E, Komulainen K, Nykänen I, Hartikainen S, Tiihonen M, Suominen AL. (2019) [42] - Finland	Non randomised population study with control and intervention	Home care	intervention group, *n* = 119, Mini-nutritional assessment (MNA) of < 24. Control group, *n* = 97, MNA < 24.	Nutrition, Oral Health and Medication – NutOrMed – Xerostomia intervention – targeted intervention for patients reporting occasional or continuous dry mouth. Written information about importance of saliva and symptoms and causes of dry mouth. Individual instructions on moistening their mouth, to use xylitol tablets or chewing gum, or dry mouth products. Advice to spread cooking oil on mucosal surfaces. Tailored nutrition intervention based on MNA score and information on plasma albumin level and 24-hour dietary recalls. Nutritionist developed a care plan to increase number of meals, energy and protein intake. Also aimed to increase liquid intake when necessary. Main goal is to correct dietary insufficiencies and make food substitutions. Patients also received written information on increasing energy and protein, good sources of protein and calcium, and good nutrition for older adults. Instructions given to participants and / or caregiver or home care nurse.	Participants given baseline examination and interview. Then six months later, data collection was repeated. Caregiver or nurse was interviewed if client was not able to reply to the interview questions to cognitive impairment.	Xerostomia (single item question – does your mouth feel dry – ‘no’, ‘occasionally’, continuously’), problems biting, problems swallowing, malnutrition or risk of malnutrition (measured using MNA < 24). Daily eating and drinking, measured as < 3 warm meals, < 2 portions of fruits or vegetables, <=5 glasses of liquids.	Restructured control and intervention groups to combine control with nutrition only intervention group – comparing xerostomia and nutrition intervention group (*n* = 66) with intervention (*n* = 138).	Feeling of dry mouth decreased by 30.3% in xerostomia intervention group vs increase of 8.7% in no xerostomia intervention group. Also reported larger decrease in malnutrition or risk of malnutrition for this group. Comparisons. Decrease in xerostomia in those suffering from occasional xerostomia was 27.3%. Drinking more than 5 glasses per day increased by 19.1% and eating three or more warm meals increased by 8.1%..	None reported.
Tuulainen, E., Nihtila, A., Komulainen, K., Nyaken, I., Hartikainen, S., Tiihonen, M., Suominen, A.L. (2020) [43] – Finland	Non randomised population study with control and intervention	Home care	231 home care clients. 75% female. 89% classified as “frail”.	Nutrition, Oral Health and Medication – NutOrMed – A nutrition intervention and an individually targeted oral health-related preventive intervention was carried out according to individual needs of the participants in the intervention group. This intervention was composed of written and oral instructions about denture hygiene, dental hygiene and cleaning of the oral mucosa, which were given to the participant or to the caregiver or nurse.	Compared control vs intervention, and “frail” vs “non frail” within these two groups. Baseline and six-month follow-up.	Oral cleaning (toothbrushing and denture cleaning frequency) and clinically determined oral hygiene (occurrence of plaque) and health (number of teeth, presence of dentures, decayed teeth and bleeding on probing)	Compared intervention outcomes for frail and non-frail participants using chi square and Mann Whitney U analyses. Multivariate logistic regression analyses to examine association between being frail score in oral health measures	A modest positive change (5.6%) in toothbrushing frequency was observed in frail intervention patients, with an 11.1% decrease in control group. Larger benefits for good denture hygiene (16.5% increase) in frail intervention group than in control group (2.3%). Larger increase in denture cleaning for control group (9.3%) than for intervention group (3.1%). Logistic regression: Frail clients had lower odds of toothbrushing and denture cleaning at baseline, higher odds for females and those using large numbers of drugs. At follow-up, frail clients still had lower odds of toothbrushing and denture cleaning, but this was no longer significant. Intervention group had higher odds to brush teeth or clean dentures. For toothbrushing those using higher numbers of drugs and for cleaning dentures those having a higher number of teeth were more likely to clean their teeth or dentures at least twice per day. No significant associations between clients classified as frail at baseline and having plaque in ≥ 20% of teeth or being edentulous, either at baseline or at follow-up.	None reported.
Wu, S.J., Wang, C.C., Kuo, S.C., Shieh, S.H. and Hwu, Y.J [44]. (2020, Taiwan)	Concurrent triangulation mixed-methods	Home care	25 nurses, 27 nursing assistants, 28 administrators and social workers, working in home & community (80%) or care institutions services (20%)	Oral hygiene education program. One day (8 hour) oral healthcare teaching established by Taiwanese Ministry of health and welfare. Four hours of presentations on relationship between oral health and general health in older adults and preventing oral diseases. One hour presentation on oral care using dental models. One hour of demonstrations of oral hygiene skills. Two hours of ‘teach-back’ technique of oral hygiene skills and a skills test of brushing and flossing.	Pre & post assessment to assess oral hygiene knowledge, attitudes and skills. Three month follow-up survey of practical application. N = 6 participants took part in 8-10 minute phone interview about changes to daily practice since the education.	Oral hygiene knowledge, attitude and skills, changes to practice since receiving the program (quantitative). Practicability of the program to improve long term care services (qualitative)	Pre-post quantitative results analysed using paired t-tests and Cohen’s d effect sizes. Qualitative data reported as summaries under thematic headings. No detail given on how themes were determined.	Less than one fifth of participants had previously received oral hygiene training. Significant improvements in pre-program and post-program oral health knowledge and attitude (measured on day of education program). Significant increase in use of oral hygiene skills (based on the oral cleaning products used; the sites receiving oral cleaning; and the frequency of performing daily oral cleaning for clients) from pre-program to three months post-program. Qualitative data suggests improvements in: clarifying misconceptions of oral health, improved staff experience (i.e. a positive experience at delivering oral health care) and identifying the importance of oral health care, especially in clients with dysphagia. Revealed a lack of oral health care training prior to the intervention and implications of oral health for other conditions such as aspiration pneumonia.	None reported.

### Setting and intervention type

The interventions in the studies included in this review fall into two broad categories –health visiting and home care. All reported interventions involved registered nurses, although it should be noted that three studies report intervention delivery involving both nursing assistants and registered nurses [39, 40, 44]. This means that the populations fall into two broad categories – children and older people. Both the health visiting and the home care study categories are split between interventions which aimed to educate patients and interventions which aimed to educate community nurses. Two of the three health visiting studies [36, 37] focused on interventions which provided oral health education to parents or primary caregivers. These interventions focused on improving nutrition, oral health behaviours, and provision of appropriate toothbrush and toothpaste. The Brickhouse et al. [36] paper also involved nurses applying a fluoride varnish to children’s teeth. The Haber et al. [38] paper focused on nurse education. The ‘Cavity Free Kids’ evidence-based curriculum was designed to increase oral health practice in health-visitor nurses.

Two of the remaining six home care papers [39, 40] are focused on the Better Oral Health in Home Care (BOHHC) intervention. This intervention aimed to improve home care workers’ knowledge of oral health, oral health assessment usage and care planning, and when it was appropriate to implement a referral to a dentist. Although this intervention was not specifically targeting registered nurses, both papers refer to registered nurses being amongst those who used the BOHHC intervention. The Wu et al. paper reports an intensive, single day, 8-hour program with 4 hours of “narration” or talks on the importance of oral health, implications for general health and prevention and treatment of disease, 2 hours of techniques and demonstrations of oral hygiene techniques, and 2 hours of “teach-back” and testing of the oral hygiene skills [44]. The other three papers [41–43] investigated the NutOrMed intervention in Finland. These papers are concerned with minor variations of this intervention, but the broad approach is a combination of dietary and oral health education and treatments for patients receiving home care. All three papers delivered tailored nutrition and oral health advice to patients, but one [41] also included a nutritional intervention for patients at risk of malnutrition and the another [42] was specifically focussed on patients receiving additional education and treatment for xerostomia. The NutOrMed intervention involves nutritional advice on healthy foods, increasing liquid intake, increasing the number of hot meals, oral health behaviours such as brushing and cleaning of the oral mucosa, and specific dry mouth strategies such as topical treatments, xylitol tablets or chewing gum. The education components of NutOrMed were delivered to the patient or to the caregiver if appropriate.

### Patient outcomes

Overall, there is a wide variability in the types of patient outcomes reported in the included studies. Both of the two health visiting papers, which educated parents, showed promising outcomes for children [36, 37]. The main outcome was that the intervention groups showed higher levels of visiting a dentist for treatment than the control. In addition, the intervention groups in these studies also showed lower levels of oral disease than the control. In the most recent paper that focused on health visitors [38], parents from the intervention group reported having received more oral health support compared to those receiving normal care. Families who ‘graduated’ the oral health program in this paper completed a survey in which none of the children showed any visible plaque, staining or decay on their child’s teeth (signs of early childhood cavities), although this comparison did not include a control group.

Turning to the papers on home care, of the two on BOHHC (the Australian home care worker education intervention), only one paper [39] looked at patient reported outcome data, measured by the OHIP-14 [45] which measures oral health related quality of life. They found increased self-reported oral health in patients of the home care workers who were in the intervention group, but with high levels of missing data these results were considered inconclusive. In the three studies of the NutOrMed intervention [41–43], improvements were found in xerostomia symptoms in comparison with control groups, but one paper [41] claimed that topical therapies were needed to achieve this, and that dietary advice alone was insufficient (although analysis was not presented to fully justify this). Positive outcomes were also reported for toothbrushing frequency and dental hygiene in “frail” patients within the intervention group. Using a logistic regression model, one paper [43] found that “frail” older people were significantly less likely to brush their teeth or clean their dentures at baseline, but post-intervention, this was no longer significant for the intervention group of “frail” older people. This suggests that the older people’s dental health habits were significantly improved using an intervention which provided them or their carers with oral health education. The sub-grouping analysis based on level of frailty is less informative for this review and it must be considered in the context of increasing numbers of analyses and sub-groups increases the risk of false positives. However, it may point to an interaction between levels of frailty and increased likelihood of poor oral health care, further underlining the need for intervention amongst this population. Only qualitative data from the care workers is reported for patient outcomes in the Wu et al. study [44], in terms of them reporting that patients are happier and appreciative of their oral care. Little concrete direction for future patient outcome assessments in interventional research in this field can be gleaned from this finding, but it does point to positive engagement from older people with the process of receiving an increased level of oral health care from nurses in their own home.

### Change to practice

Only one study within the health visiting papers reported analysis of change to practice [38]. They found that nurses who participated in an oral health education program showed higher levels of positive oral health behaviours and practices, such as explaining toothbrushing techniques and how to prevent cavities to parents and improved levels of dentist referrals. For the home care papers, only the BOHCC intervention studies reported analyses of changes to practice [39, 40]. Home care workers reported significantly improved oral health knowledge and skills after participating in the BOHCC intervention and this increased staff ability to recognize patients in need of oral health support and dental referrals [40]. Prior to receiving an intervention, there was no evidence of oral health assessments by home care workers across four different home care provision organisations in one study [39], but this increased post-intervention. Oral health care planning was also successfully increased, alongside staff confidence on carrying out oral health care with their patients. The Wu et al. study reports significant increase in nurses’ and nursing assistants’ use of oral hygiene products and oral hygiene techniques in daily practice, assessed 3 months post-intervention [44]. They also reported significantly improved oral health knowledge and skills, assessed immediately post-intervention.

### Implementation and process evaluation

Only one of the health visiting papers explicitly reported any implementation analysis: 100% of nurses who received support to use the intervention were still using it after ninety days, compared with only 30% of nurses who did not receive support [38]. Whilst remaining aware of the small sample size (*n* = 32 nurses), this is evidence that an educational intervention, without professional support to use it, is considerably less likely to be maintained and used than an intervention delivered alongside professional support. The paucity of data available in this area is indicative of very limited research into what is a critical aspect of intervention design, that is the accurate measurement of facilitators and barriers to successful intervention delivery and to changing practice.

Other studies reported some contextual findings, despite not categorising this explicitly as implementation or process findings. Home care workers reported experiencing a ‘lone worker’ feeling prior to practice changes in one study [39]. This was improved by promoting a “stop, check and act” strategy where they were taught to identify changes in oral health and report to care-coordinators. Building relationships between home care clients and staff, respect for cultural traditions of clients, and close mentoring from the project director were all seen as facilitators for the intervention in this study. It was also found that building the BOHHC model into daily care procedures and developing dental referral pathways were seen as facilitators. A knowledge transfer expert was also found to increase ‘engaged scholarship’ of project management staff, who themselves were then able to mentor care staff. Similar facilitators were found in the other study of BOHHC in terms of corporate engagement and capacity building networks [40]. High staff turnover was found to be a barrier to intervention usage. Although process evaluation is not reported in the Wu et al. paper, they do report that less than one fifth of their 80 participants (the majority of which were nurses or nursing assistants) had received prior training on oral health care, further underlining the minimal prior level of training in this field and the context in which oral health education interventions are likely to be delivered [44].

## Discussion

This scoping review aimed to inform an oral healthcare intervention for nurses caring for people living in community settings. We permitted interventions for health visitors to be included, as information on changing practice in this setting was likely to have transferability. Even looking across both these fields of nursing, there was a limited number of papers on oral health interventions in the community. This indicates a huge gap between the important role which nurses can play in the provision of oral health care at home and the number of research studies conducted in this field.

Overall, there is some evidence for patient, caregiver, parent and nurse knowledge of oral health being successfully improved through education interventions. The design of such education interventions for nurses in other community settings, such as care homes, has been found to be effective when a co-design methodology is used, involving collaborative efforts between researchers and nurses and ensuring that the complexity of the system in which the intervention will be used is considered [46]. The scarcity of implementation and process analyses in the papers included in this review may be representative of how traditional evaluations can underestimate or underrepresent the complexity of the system surrounding intervention delivery. This can be ameliorated using a participatory design approach to interventions, ensuring full consideration of the system in which the intervention is being delivered [47].

The evidence of efficacy in health visitor settings is mostly promising. The wide range of oral health measures in the included studies is not conducive to making a strong claim about the effectiveness of community nurse-led interventions for oral health. However, there is a generally positive pattern of patient outcomes, suggesting that this realm of intervention has promise and warrants further study. These outcomes also reveal that patterns of oral health habits can be improved by educating older people about oral health. This suggests that any future intervention development would benefit from not only building capability in nurses themselves, but also ensuring that information about self-care is provided to older people and/or their carers where possible. The vulnerable population in one of the health visitor studies may have some transferable learning for older people and patients with complex needs in the community: that nurse-led interventions can improve the basics of oral health care for vulnerable groups, and this can improve patient outcomes. The evidence for efficacy in home care settings is stronger. More importantly than the overall efficacy, the nature of the intervention and associated success reveals important components of successful interventions. The NutOrMed studies show considerable success in improving oral health care in older people with a variety of specific subgroups (e.g., those with xerostomia and “frail” patients) using a combined nutritional and oral health care approach. Frailty is a medical condition that affects older people and their ability to recover from adverse health related events including falls, disability, institutionalisation, cognitive impairment and death. More recently, several empirical studies have determined a strong association between oral health and frailty. A recent evidence synthesis on the topic, concluded that oral health problems in older age are likely to be a risk factor for a frailty syndrome [48]. Interventions that improve oral health amongst older people living with frailty at home are therefore very useful for community nurses. Educating patients and caregivers, alongside nutritional interventions show some evidence of successfully improving oral health. This may be helpful in the design of oral health interventions, that nutrition and lifestyle factors should be considered, not just specific oral health care techniques.

The studies in this review show that changes to community nursing practice are possible with education interventions for staff and can result in increased capability to make dental referrals, conduct oral health assessments and educate patients on oral health care. This is considerably more likely to be successful when implementation factors which provide professional support are present. Education without some form of support to change practice is unlikely to be implemented sustainably. This includes support from management, mentoring and procedural change (e.g., the stop, check and act process). This points to the importance of the adoption of established frameworks for implementation when engaging in interventional research in this field [49], yet the absence of this is starkly apparent in the studies found by this review. It is important to acknowledge that although many of these studies report statistically significant findings for changes to practice, this does not necessarily reflect improvements which are clinically significant. For example, a significant increase in use of oral hygiene products does not offer a comprehensive answer to the question of whether oral health care was improved to an extent that would be clinically beneficial.

A further barrier in the Lewis et al. study was high rates of staff turnover in home care nursing [40]. This provides a significant challenge to any attempts to promote sustainable change to practice. Such a barrier could potentially be tackled by the incorporation of digital learning, ensuring that any education intervention can be delivered online and reduce staff time demand to introduce the training to new staff. This review has highlighted both the significant lack of prior training found in some settings (e.g., < 20% in Wu et al. study [44]) and the need for institutional support to maximise effectiveness. It should be considered that in the UK, NICE have made several recommendations for oral health in care home settings (NG48). This clinical guidance promotes the importance of oral health assessments, person-centred care planning, daily mouth care and any strategies that can improve knowledge about oral health care for care home staff [50]. While this clinical guidance is important, there is less explicit guidance for older people receiving care in their own home despite the potential for similarity between these populations in terms of oral health risks.

An additional contextual issue for interpreting these findings is that a significant portion of home care is delivered by care workers who are not registered nurses. Three of the papers [39, 40, 44] did not exclusively involve registered nurses, and therefore, it appears that capability building for a wider range of care workers is possible and not just for registered nurses. It may also be the case that improving the oral health care skills and knowledge of registered nurses will allow further dissemination within the community, e.g. to family carers and to other community services for whom nurses typically act as gatekeeper.

## Conclusions

Overall, this review has highlighted that despite the plethora of evidence for the impacts of poor oral health and the likelihood that people who receive care in their own homes will struggle to receive oral health care, there is a paucity of interventional research in this area. The small number of interventions for oral health in community nursing, and even smaller number of education interventions for nurses themselves is a significant issue and underlines the need to direct these findings into practical application as effectively as possible. It is clear from this review that oral health interventions for community nurses show promise for both change to practice and patient outcomes, but that barriers such as time pressure and high staff turnover must be considered. Therefore, streamlined and digital education for oral health care may have a high potential for successfully building the necessary capability in community nurses. An online, digital approach may also reduce the demand on staff time and allow rapid capability building in new staff.

## Data Availability

All data generated or analysed during this study are included in this published article.
